# Association Between VACS Index and Health-Related Quality of Life in Persons with HIV: Moderating Role of Fruit and Vegetable Consumption

**DOI:** 10.1007/s12529-022-10096-4

**Published:** 2022-05-09

**Authors:** Laura M. Campbell, Jessica L. Montoya, Pariya L. Fazeli, Maria J. Marquine, Ronald J. Ellis, Dilip V. Jeste, David J. Moore, Raeanne C. Moore

**Affiliations:** 1 SDSU/UC San Diego Joint Doctoral Program in Clinical Psychology, San Diego, CA USA; 2 Department of Psychiatry, University of California San Diego, 220 Dickinson Street, Suite B (8231), San Diego, CA 92103 USA; 3 Department of Family, Community, and Health Systems, School of Nursing, University of Alabama at Birmingham, Birmingham, AL USA; 4 Department of Neurosciences, University of California, San Diego, San Diego, CA USA; 5 Sam and Rose Stein Institute for Research On Aging, University of California, San Diego, CA USA

**Keywords:** Behavioral health, Infectious disease, Wellbeing, Healthcare, Outcome assessment, Prevention

## Abstract

**Background:**

The health status of people with HIV (PWH) influences their health-related quality of life (HRQOL). Modifiable lifestyle factors may improve HRQOL. This study (1) explores the association between modifiable lifestyle factors (physical activity and nutrition) and HRQOL and (2) examines if these lifestyle factors moderate the association health status and HRQOL.

**Methods:**

Participants included 91 community dwelling PWH (age 36–65 years) from the university lab. Participants reported mental and physical HRQOL via the Medical Outcome Study 36-Item Short-Form (SF-36). Physical activity was examined via the International Physical Activity Questionnaire and nutrition (i.e., fruit and vegetable consumption) was assessed with the By-Meal Screener. Health status was ascertained via the Veterans Aging Cohort Study (VACS) Index.

**Results:**

Aim 1 analyses indicated that neither physical activity nor nutrition was related to mental HRQOL (*p*’s > .05). However, greater physical activity (β = .33, *p* < .01) and nutrition (β = .23, *p* = .03) were each independently related to better physical HRQOL and remained significant after accounting for co-occurring medical conditions. For aim 2, the interaction between health status and nutrition was statistically significant (β = .24, *p* = .02), such that the association between worse health status and worse physical HRQOL was weaker with better nutrition. There was not a statistically significant interaction between physical activity and health status on physical HRQOL (*p* > .05).

**Conclusion:**

Physical HRQOL is related to self-reported physical activity and nutrition, with nutrition showing a moderating effect on the association between health status and physical HRQOL. Thus, future interventional studies designed to improve physical HRQOL should target both physical activity and nutrition.

## Introduction

The advent of antiretroviral therapy (ART) has significantly increased life expectancy for people with HIV (PWH) [1]. With ART, HIV is now a long-term chronic disease, and there has been increased focus on examining determinants of health-related quality of life (HRQOL) among PWH. HRQOL has been shown to be significantly lower in PWH than the general population [2]. PWH are at greater risk for several medical (e.g., diabetes) and neuropsychiatric comorbidities (e.g., depression, apathy) that are negatively associated with HRQOL [3–6]. Among PWH, better HRQOL is associated with a number of favorable outcomes such as better ART adherence, higher rates of employment, and successful cognitive aging [3, 7]. Therefore, it is important to understand modifiable factors that may promote better HRQOL among PWH.

Physical activity is one modifiable lifestyle factor that has been repeatedly associated with better physical and mental HRQOL among PWH in observational research [8–11]. Additionally, interventions aimed at increasing physical activity among PWH have also shown that HRQOL improves with increased physical activity [12, 13]. However, other modifiable lifestyle factors, such as nutrition, have been less explored in the context of HIV disease. Malnutrition and food insecurity have been associated with worse quality of life [14, 15]. To date, we are aware of only one observational study among PWH that has examined the relationship between nutrition and HRQOL. This study found better nutrition was significantly associated with better HRQOL, specifically general health, physical health, and mental health [16]. Thus, both physical activity and nutrition are modifiable lifestyle factors that may influence HRQOL. Moreover, there is some literature to suggest that targeting multiple lifestyle interventions together (e.g., physical activity and nutrition) may be more efficacious in some populations (e.g., frail older adults) than targeting one at a time [17]. Promotion of healthy lifestyle factors, such as physical activity and nutrition, is a recommended component of comprehensive care for PWH [13], yet combination interventions have not been well studied in PWH. Gaining a better understanding of multiple modifiable lifestyle factors in PWH can help in designing more efficacious interventions to improve HRQOL.

While morbidity and mortality have dramatically decreased in the ART era, PWH are still at greater risk for a number of medical and psychiatric comorbidities (e.g., diabetes, HCV, depression) that may impact HRQOL [4, 18]. Both physical activity and good nutrition have been shown to reduce the risk of cardiovascular and metabolic conditions, and these comorbid conditions have been shown to adversely affect HRQOL in PWH, particularly physical HRQOL [19–21]. Moreover, comorbidity burden has been shown to negatively impact HRQOL among PWH [22], with some indication that medical factors may impact HRQOL in PWH even more so than people without HIV [23]. Overall, there is evidence that lifestyle factors such as physical activity and nutrition are positively associated with HRQOL and health status and comorbid conditions are negatively associated with HRQOL; however, an under-explored area of research is whether modifiable lifestyle factors may moderate the relationship between poor health status and worse HRQOL among PWH.

Building from prior literature, the objectives of this study were to examine the relationship between modifiable lifestyle factors (physical activity and nutrition) and HRQOL among PWH and to examine whether these modifiable lifestyle factors moderate the association between poorer health status and worse HRQOL. Aim 1 is to investigate the association of physical activity and nutrition with mental and physical HRQOL. We hypothesize that greater self-reported physical activity and better nutrition would synergistically interact with one another to impact (positively) mental and physical HRQOL. Aim 2 is to examine if physical activity and nutrition moderate the relationship between poorer health status (as measured by the Veterans Aging Cohort Study (VACS) index) and HRQOL. We hypothesize that the relationship between poorer health status and worse HRQOL would be weaker with greater self-reported physical activity and better nutrition. A greater understanding of the degree to which modifiable lifestyle behaviors may influence the relationship between health status and HRQOL may inform intervention strategies to promote better HRQOL in PWH.

## Methods

### Participants and Design

This study examined cross-sectional baseline data from 91 community dwelling PWH from the longitudinal observational *Multi-Dimensional Successful Aging among HIV-Infected Adults* conducted at the University of California San Diego. Participants were recruited from the broader San Diego community as well as from ongoing studies at the HIV Neurobehavioral Research Program. The study was approved by the University of California San Diego institutional review board, and all procedures performed in studies involving human participants were in accordance with the ethical standards of the University of California San Diego and with the 1964 Helsinki declaration and its later amendments. All participants completed and passed the UCSD Brief Assessment of Capacity to Consent [24] and provided written, informed consent. Inclusion study for this study included having HIV disease (> 3 years), being primarily English speaking, and being between the ages of 36 and 65. Exclusion criteria for the study were minimal and included: serious mental illness with psychotic features, significant neurological conditions (e.g., stroke), and positive urine toxicology (excluding marijuana and prescription medications) on the day of testing. Additionally, participants were excluded from all analyses if they did not have available or valid data (as described below) for physical activity, nutrition, HRQOL, or VACS variables. Ninety-one PWH met these requirements and were included in this study.

### Measures

## Health-Related Quality of Life

HRQOL was measured with the Medical Outcome Study 36-Item Short-Form (MOS-SF-36) [25], which measures eight health concepts with greater scores indicating better HRQOL. The MOS-SF-36 has been widely used in HIV studies, and has demonstrated reliability and validity in PWH [26, 27]. For this analysis, the two main summary scores, physical HRQOL and mental HRQOL, were used as primary outcome variables.

## Modifiable Lifestyle Behavior—Physical Activity

The International Physical Activity Questionnaire (IPAQ) Short Form [28] was administered to assess self-reported physical activity. Participants reported frequency (i.e., number of days) and time (i.e., minutes) spent engaging in walking, moderate-intensity activities, and vigorous-intensity activities over the past 7 days. Per IPAQ scoring guidelines, reported minutes at each activity level were multiplied by metabolic equivalents (MET; i.e., walking: 3.3, moderate: 4.0, and vigorous: 8.0), multiplied by the reported frequency, and summed to obtain total MET minutes/week. We will refer to total MET minutes/week as “physical activity” in the “Results” section. As recommended by IPAQ guidelines, participants with reported total physical activity that was greater than or equal to 16 h/week (*n* = 2) were considered invalid and thus excluded from the study.

## Modifiable Lifestyle Behavior—Nutrition (i.e., Fruit and Vegetable Consumption)

The National Cancer Institutes By Meal Screener [29] was used to measure self-reported daily fruit and vegetable consumption. Participants were queried about frequency and quantity of fruits and vegetables consumed during the day (i.e., morning, afternoon, and evening) over the past month. Using the National Cancer Institute’s guidelines, fruit and vegetable servings were first converted to USDA’s 2005 MyPyramid serving equivalents, and the frequency was converted to average number of times consumed per day [30]. Frequency and servings were multiplied and then summed to get total daily fruit and vegetable consumption which is referred to as “nutrition” in the “Results” section. In line with the original By Meal Screener publication by Thompson et al. (2002), participants were excluded from this study if, based on this sample, estimated daily consumption was two interquartile ranges below the first quartile (*n* = 0) or above the third quartile (*n* = 2).

## VACS Index

The Veteran Aging Cohort Study Index (VACS Index) is a composite biomarker of disease severity among PWH that has been associated with mortality [31, 32] and other negative health outcomes (e.g., ICU admission, frailty, cognitive impairment) [33–36]. The VACS Index is a composite that includes age, HIV biomarkers (i.e., HIV plasma RNA, current CD4 count), and markers of common medical comorbidities in HIV (i.e., renal function [glomerular filtration rate; eGFR], liver function [fibrosis index; FIB-4], anemia [hemoglobin], and HCV co-infection) [37, 38]. In 2019, the VACS Index 2.0 was validated and incorporates albumin, white blood cell count, and body mass index as these variables were found to improve discrimination of mortality in PWH [39]. Higher scores on the VACS Index and VACS Index 2.0 are indicative of worse health status. The original VACS Index is used in the majority of analyses in this study as some individuals were missing albumin, white blood cell count, and/or BMI to create the VACS Index 2.0; however, sensitivity analyses were re-run with a smaller sample size to assess possible differences found via the VACS Index 2.0 (*n* = 80).

### Medical and Psychiatric Assessment

Participants underwent a standardized neuromedical evaluation to assess relevant comorbidities. Presence of medical comorbidities in Table 1 was determined by self-reported diagnosis and/or taking medications for the condition except for BMI, which was calculated from measured height and weight. The Composite International Diagnostic Interview (CIDI v2.1) [40], a computer-assisted, fully structured interview, was used to diagnose DSM-IV lifetime and current affective and substance use disorders. All participants underwent a test for HIV and HCV infection using an HIV/HCV finger stick point of care test (Abbott RealTime HIV-1 test, Abbott Laboratories, Illinois, USA). AIDS diagnosis, Nadir CD4 + T-cell count, estimated duration of HIV disease, and ART regimen were obtained via self-report. Current CD4 + T-cell count and HIV RNA level were measured in blood plasma in a CLIA-certified lab.

**Table 1:** Participant characteristics (*n* = 91)

	M (SD), median [IQR], *n* (%)
*Demographic variables*	
Age (years), M (SD)	51.3 (8.2)
Male, *n* (%)	77 (85%)
Race/ethnicity	
Non-Hispanic White	50 (55%)
African American/Black	17 (19%)
Hispanic/Latino	16 (18%)
Other	8 (8%)
Education (years), M (SD)	14.1 (2.5)
Not employed (unemployed, retired, disability), *n* (%)	60 (66%)
*Comorbid conditions*	
Hyperlipidemia, *n* (%)	37 (41%)
Hypertension, *n* (%)	43 (47%)
Diabetes mellitus, *n* (%)	9 (10%)
Hepatitis C, *n* (%)	15 (16%)
BMI^a^, *M* (SD)	27.5 (5.6)
VACS Index, *M* (SD)	19 (12.5)
VACS Index 2.0^b^, *M* (SD)	39 (10.9)
LT MDD^a^, *n* (%)	46 (53%)
Current MDD, *n* (%)	9 (10%)
LT any substance use disorder^a^, *n* (%)	58 (67%)
Current substance use disorder, *n* (%)	3 (3%)
*HIV characteristics*	
AIDS, *n* (%)	56 (62%)
Current CD4, median [IQR]	629 [432–853]
Nadir CD4, median [IQR]	180 [52–300]
Duration of HIV infection (years), median [IQR]	19.0 [11.0–24.7]
On ART^c^, *n* (%)	88 (98%)
Undetectable viral load, *n* (%)	84 (92%)
*Health-related quality of life*	
Physical	45.6 (12.4)
Mental	46.3 (14.5)
*Lifestyle factors*	
Daily fruits and vegetable servings, median [IQR]	2.6 [1.4, 4.0]
Total physical activity, median [IQR]	1386 [120, 4746]

Total physical activity = daily MET min/week

*BMI*, body mass index; *VACS Index*, Veteran’s Aging Cohort Study Index; *LT*, lifetime; *MDD*, major depressive disorder; *ART*, antiretroviral therapy

^a^*n* = 86

^b^*n* = 80

^c^*n* = 90

### Statistical Analyses

JMP version 14.0.0 was used for statistical analyses. Spearman rho analyses were used to examine the univariate relationship between physical activity and nutrition and physical and mental HRQOL. Model 1 (corresponding with Aim 1) examined the relationship of physical activity, nutrition, and their interaction on physical HRQOL using a multivariable linear regression. Model 2 examined the relationship of physical activity and nutrition with physical HRQOL after accounting for VACS Index and covariates. Model 3 (corresponding with Aim 2) examined a VACS by physical activity interaction and a VACS by nutrition interaction on physical HRQOL. For Models 2 and 3, which included VACS Index and relevant covariates, a stepwise multiple regression model using backward selection based on Akaike information criterion (AIC) was used. The VACS Index and demographic variables, comorbid conditions, and HIV disease characteristics not already incorporated into the VACS Index were screened as potential covariates. These potential covariates are included in Table 1. Potential covariates that were associated with physical HRQOL at *p* < 0.10 (i.e., diabetes, hypertension, hyperlipidemia, sex, and current major depression; BMI was not included due to missing data but is included as part of the VACS Index 2.0 which was also explored in follow-up analyses) were entered into the model and retained as covariates in the models if they improved the overall optimal AIC model due to their significant contributions to the overall model fit. In a subsample with available data to generate VACS Index 2.0, Models 2 and 3 were re-run using the VACS Index 2.0 instead of the original VACS Index.

## Results

Participants were between the ages of 36 and 65, with a mean age of 51.3 (SD = 8.2). Majority of participants were men (85%), and approximately half (55%) were non-Hispanic white. The majority were on ART (98%) and had undetectable viral load (92%; < 50 copies/mL in plasma). Demographic and clinical characteristic of the sample are included in Table 1.

### Modifiable Lifestyle Behaviors (Physical Activity and Nutrition) Are Associated with Health-Related Quality of Life

In univariate analyses, physical activity (*ρ* = 0.42, *p* < 0.001) and daily nutrition (*ρ* = 0.39, *p* < 0.001) were significantly correlated with physical HRQOL such that greater physical activity and better nutrition (i.e., greater consumption of fruits and vegetables) were both associated with better physical HRQOL. Neither physical activity (ρ = 0.17, *p* = 0.11) nor nutrition (ρ = 0.13, *p* = 0.22) was significantly related to mental HRQOL; therefore, mental HRQOL was not examined in subsequent analyses.

### Modifiable Lifestyle Behaviors (Physical Activity and Nutrition) Do Not Show a Synergistic Interaction on Physical Health-Related Quality of Life

**Fig. 1:**
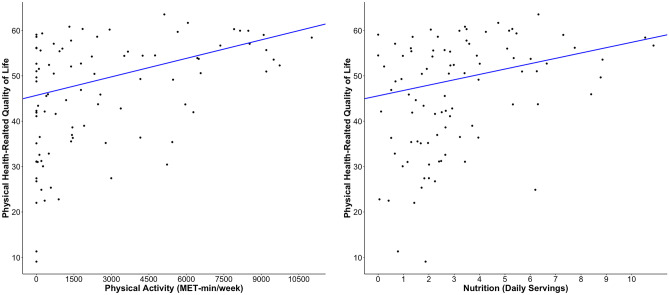
The relationship between physical activity and physical health-related quality of life (left) and nutrition and physical health-related quality of life (right). *Note.* MET, metabolic equivalents; nutrition, daily fruit and vegetable servings

In multivariable analyses, the physical activity by nutrition interaction was not significant (*β* = − 0.14, *p* = 0.19) and thus removed from the model. After removing the interaction term, greater physical activity (*β* = 0.33, *p* = 0.002) and nutrition (*β* = 0.23, *p* = 0.031) were significantly and independently related to better physical HRQOL. Residuals were somewhat skewed, so the model was re-run by transforming physical HRQOL (squared); results did not significantly change so non-transformed results are presented to aid in interpretation. See Table 1 for Model 1 estimates and Fig. 1 for the association between physical activity and nutrition with physical HRQOL.

When adding the VACS Index to the model as well as other covariates that significantly improved model fit (i.e., diabetes and hyperlipidemia), physical activity (*β* = 0.33, *p* < 0.001) and nutrition (*β* = 0.20, *p* = 0.044) remained significantly positively associated with physical HRQOL. Additionally, VACS Index was negatively associated with physical HRQOL (β = − 0.25, *p* = 0.004) such that higher VACS Index scores (indicative of worse health) were associated with worse physical HRQOL. See Table 2 for Model 2 estimates.

**Table 2:** Linear regressions to examine the relationship between physical activity and nutrition with physical health-related quality of life

Variable	Estimate	SE	95% CI	Std. Estimate	T	*p* value
*Model 1:*						
Physical activity	0.001	0.0004	[0.0005, 0.002]	0.33	3.22	0.002
Nutrition	1.18	0.54	[0.11, 2.25]	0.23	2.18	0.031
*Model 2: (with covariates)*						
Physical activity	0.001	0.0004	[0.002, 0.334]	0.33	3.46	< 0.001
Nutrition	1.03	0.50	[0.028, 2.03]	0.20	2.04	0.044
VACS Index	−0.26	0.09	[−0.43, −0.08]	−0.25	−2.94	0.004
Diabetes	−6.43	3.79	[−13.98, 1.10]	−0.16	−1.70	0.093
Hyperlipidemia	−3.48	2.26	[−7.98, 1.02]	−0.14	−1.54	0.128
*Model 3: (VACS by nutrition interaction)*						
Physical activity	0.001	0.0004	[0.0005, 0.002]	0.31	3.29	0.001
Nutrition	1.25	0.51	[0.24, 2.26]	0.24	2.50	0.016
VACS Index	−0.22	0.09	[−0.40, −0.05]	−0.22	−2.55	0.013
Nutrition*VACS Index	0.11	0.05	[0.014, 0.21]	0.21	2.28	0.025
Diabetes	−6.80	3.64	[−14.04, 0.45]	−0.16	−1.87	0.066

Physical activity, MET min/week; Nutrition, daily fruit and vegetable servings; *VACS Index*, The Veterans Aging Cohort Study Index

### The VACS by Nutrition Interaction Is Significantly Associated with Physical Health-Related Quality of Life

**Fig. 2:**
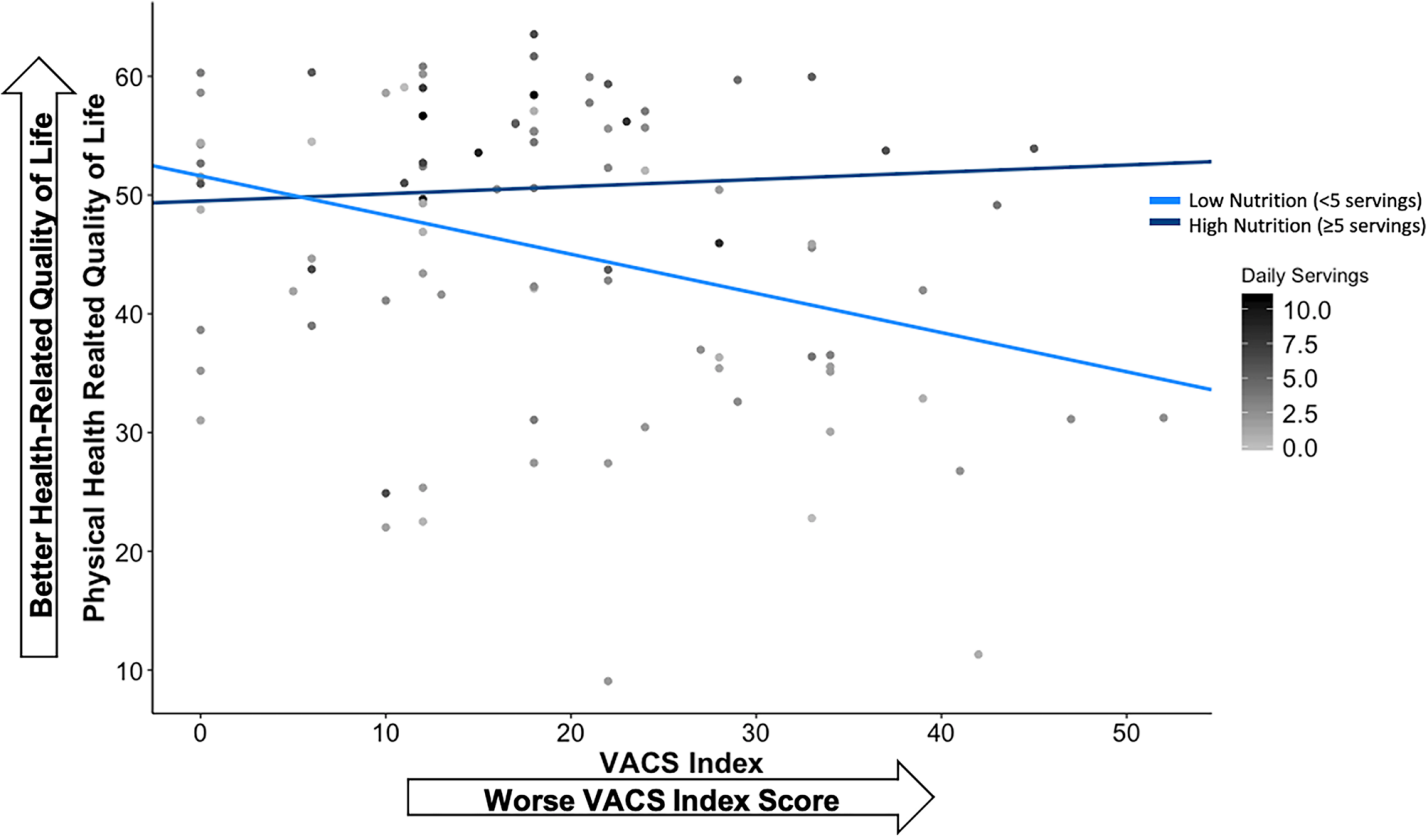
Nutrition moderates the negative relationship between VACS Index and physical health-related quality of life. *Note.* Model was run with nutrition as a continuous variable but was dichotomized into low (< 5 daily servings) and high (≥ 5 daily servings) nutrition for demonstrative purposes. Regression lines adjust for other variables in the model (i.e., physical activity, diabetes). VACS Index, The Veterans Aging Cohort Study Index; nutrition, daily fruit and vegetable servings

When examining the VACS Index by lifestyle factor (i.e., physical activity and nutrition) interactions, the VACS Index by physical activity interaction did not significantly improve the model; therefore, it was not retained in the model. Diabetes status was the only covariate that was included in the model given that it was the only covariate that significantly improved model fit. The main effect of physical activity was still significant (*β* = 0.31, *p* = 0.001) such that greater physical activity was related to better physical HRQOL. Additionally, the nutrition by VACS Index interaction was significant (*β* = 0.21, *p* = 0.025), indicating that with better nutrition, the negative association between VACS Index and physical HRQOL is weaker. See Table 2 for Model 3 estimates and see Fig. 2 for a visual depiction of the nutrition by VACS Index interaction.

When examining the VACS Index 2.0, with a smaller sample size (*n* = 80), the VACS 2.0 by nutrition interaction was not significant (*β* = 0.17, *p* = 0.106). After removing the interaction, greater physical activity (*β* = 0.27, *p* = 0.013) and better nutrition (*β* = 0.22, *p* = 0.045) remained associated with better physical HRQOL. The VACS Index 2.0 was not significantly associated with physical HRQOL (*β* = − 0.12 *p* = 0.217).

## Discussion

In this cross-sectional study of middle-aged to older PWH, we found both physical activity and nutrition (i.e., fruit and vegetable consumption), assessed via self-report questionnaires, were independently associated with physical HRQOL but not mental HRQOL. Moreover, the association between these modifiable lifestyle behaviors and physical HRQOL was additive rather than synergistic (i.e., the interaction between physical activity and nutrition on physical HRQOL was not statistically significant). The association between modifiable lifestyle behaviors and physical HRQOL remained statistically significant even after accounting for other comorbidities that may impact physical HRQOL. Lastly, we found there was a significant interaction between nutrition and health status (as measured by the VACS Index), which may suggest that better nutrition buffered the association between poorer health status and worse physical HRQOL.

Our study’s finding that both self-reported physical activity and nutrition related to better physical HRQOL adds to the limited literature supporting that these health-promoting modifiable lifestyle behaviors may be beneficial in promoting better physical HRQOL. This study is also the first to our knowledge to examine if the association between these lifestyle factors and physical HRQOL is additive or synergistic. Overall, these data suggest that physical activity and nutrition may have additive effects on physical HRQOL. This finding is important because some interventions only target one modifiable lifestyle behavior [13], even though it may be beneficial to target multiple behaviors (e.g., both physical activity and nutrition). It is not surprising that both physical activity and nutrition relate to better physical HRQOL given previous studies have reported these positive associations [16]. Additionally, both physical activity and nutrition have been associated with lower risk of cardiometabolic conditions as well as lower levels of inflammation [41–43]. However, specific biological mechanisms by which physical activity and nutrition may exert a positive effect on physical HRQOL, particularly in the context of HIV, need to be further explored.

Physical activity and nutrition were both gathered through self-report questionnaires. The IPAQ has been shown to have acceptable measurement properties as compared to other self-report measures [28], and it has been shown to correlate with objectively measured physical activity among PWH [44]. However, studies have shown that adults, including PWH, overestimate physical activity on the IPAQ [28, 44] compared to objective measures of physical activity. Future studies may benefit from utilizing objective measures such as accelerometers to measure physical activity that can more objectively examine these relationships and examine which types of exercise (e.g., intensity) may be most beneficial. Moreover, the By Meal Screener questionnaire that was used to assess nutrition is not a comprehensive assessment of daily diet, and this screener has also been shown to overestimate fruit and vegetable consumption [29]. Additionally, this screener assesses total fruit and vegetable consumption; however, there is growing evidence that specific foods (e.g., foods high in polyunsaturated fats, the Mediterranean diet) may be particularly beneficial for cardiometabolic health and reducing inflammation [45]. Thus, future studies may want to utilize other measures that could more accurately measure all food consumption such as daily food diaries or ecological momentary assessment to objectively assess nutrition to lend insight into which foods may be most beneficial.

It is important to also consider that those with greater physical activity or better nutrition may be healthier to begin with. Additionally, these modifiable lifestyle behaviors may also relate to other factors not collected in this study, such as socioeconomic status (SES) and access to healthcare, which could also account for some of the observed relationships. Nevertheless, this study does provide evidence that interventional research is warranted, particularly because physical activity and nutrition interventions have been shown to be successful in promoting exercise and better nutrition among PWH [13]. In order to achieve optimal outcomes, it will be important for interventions to personalize physical activity and nutritional goals among PWH. Many PWH may have a number of medical factors (e.g., pain, neuropathy) and/or barriers (e.g., stigma, access to transportation to safe exercise) that can interfere with engagement in physical activity and need to be considered when creating physical activity goals [46]. Fortunately, research suggests that low-impact physical activity such as yoga and tai chi, which may be more appropriate for some PWH, has positive benefits such as increased quality of life and improved health [47–49].

One intriguing finding was that greater nutrition somewhat attenuated the relationship between poorer health status and worse physical HRQOL. This may suggest that good nutrition may be particularly important for PWH with poorer physical health, and this would be a particularly important group to target with nutrition interventions. There are again a number of potential barriers to a healthy diet that should be considered when designing nutrition interventions. For example, nutritious food is not easily accessible to many due to food deserts and/or food insecurity and other health considerations (e.g., gastrointestinal issues, diabetes); furthermore, physical limitations may make it more difficult to prepare food. Therefore, the goals of nutrition interventions should be collaboratively discussed rather than a “one size fits all” approach [50]. In sum, physical activity and nutrition are modifiable lifestyle behaviors that can and should be personalized, but it must be a collaborative process in order to design attainable and sustainable goals (e.g., as discussed in Montoya, Jankowski [46]).

Neither physical activity nor nutrition was significantly associated with mental HRQOL. This was unexpected given that physical activity has previously been associated with better mental HRQOL among PWH and has been associated with other psychological factors that can impact mental HRQOL such as depression [8, 10, 51]. Moreover, the only study that has examined nutrition and HRQOL found a positive relationship between nutrition and mental HRQOL [16]. There are several possible reasons why we did not observe this relationship. For example, other studies have found only specific types of exercise (e.g., aerobic or moderate/vigorous exercise) relate to mental HRQOL and depression [10, 51]. The physical activity measure used in this study does not differentiate between different types or intensity of exercise, which may have limited our ability to detect an association. Moreover, reported physical activity was fairly low in this sample again indicating we may not have had enough variability in this variable to detect an association with mental HRQOL.

There are additional limitations to this study that should be considered. First, this analysis was cross-sectional and thus precludes the ability to make causal inferences; thus, longitudinal and intervention studies are needed to better understand these relationships. Second, this study focused on the VACS Index as a marker of physiological health given that it is associated with a number of risk factors among PWH and is often used in a clinical setting. However, other studies may want to expand this research by examining other risk factors such as frailty and comorbidity burden. Third, while we considered demographic variables as potential covariates, we were unable to examine if these relationships differed by demographics (e.g., sex, race/ethnicity, age) due to limited sample size. Future observational and intervention studies may want to examine if the association between lifestyle factors and HRQOL differs by demographic factors to inform more personalized intervention strategies.

Overall, our findings indicate both physical activity and nutrition may be associated with physical HRQOL and highlight the need for observational and interventional research studies to further examine these associations. PWH tend to be less physically active than other people with chronic diseases [52] and are at increased risk for undernutrition and food insecurity [53]. As such, both physical activity and nutrition are modifiable lifestyle behaviors that could be targeted through personalized interventions which may in turn improve or sustain physical HRQOL. Personalized interventions may be particularly relevant for PWH as these interventions could be tailored to address barriers such as medical conditions that preclude more traditional high-impact exercise, limited access to transportation, and food insecurity.

## Data Availability

Data cannot be shared publicly to maintain full participant confidentiality as there is a substantial risk of reidentification of study participants. Data obtained was from a specific and vulnerable population in a specific city in the USA that could become identifying despite efforts to anonymize data. However, data are available from the HIV Neurobehavioral Research Center’s Data Management and Information System (DMIS) Committee (contact: hnrpresource@ucsd.edu) to researchers who meet the criteria for access to confidential deidentified data.
